# Endocrine-Immune Interactions in Pregnant Non-Human Primates With Intrauterine Infection

**DOI:** 10.1155/S1064744997000227

**Published:** 1997

**Authors:** Michael G. Gravett, Miles J. Novy

**Affiliations:** 1 Division of Reproductive Sciences Oregon Regional Primate Research Center 505 NW 185th Avenue Beaverton OR 97006 USA; 2 Departments of Obstetrics and Gynecology Legacy Emanuel Hospital and Health Center Portlaud Oregon USA; 3 Oregon Health Sciences University Beaverton and Portland Oregon USA

## Abstract

Preterm birth remains the most common cause of perinatal mortality. Although the causes of preterm labor are multifactorial and vary according to gestational age, preterm labor and term labor share common cellular and molecular mechanisms, including stimulation of the fetal hypothalamic-pituitary-adrenal (HPA) axis and endocrine/immune system interactions. We have developed a non-human primate experimental model for intrauterine infection and preterm labor using chronically instrumented rhesus monkeys (*Macaca mulatta*) with timed gestations. We have documented the temporal and quantitative relationships among intrauterine infection, the synthesis and release of proinflammatory cytokines, prostaglandins, and fetal-placental steroid biosynthesis in this model. Infection-induced preterm parturition is characterized by significant elevations in amniotic fluid proinflammatory cytokines and by increases in fetal adrenal steroid biosynthesis, but not by corresponding increases in placental estrogen biosynthesis characteristic of spontaneous parturition. This suggests that activation of the fetal HPA axis by the stress of infection is accompanied by placental dysfunction and also that infection-induced preterm parturition is not dependent upon the increased estrogen biosynthesis observed in spontaneous parturition. These different endocrine and immune responses have important diagnostic and therapeutic implications in the management of preterm labor.


Infectious Diseases in Obstetrics and Gynecology 5:142–153 (1997)
© 1997 Wiley-Liss, Inc.

### Endocrine-Immune Interactions in Pregnant Non-Human Primates With Intrauterine Infection

**Michael G. Gravett and Miles J. Novy**

*Division of Reproductive Sciences, Oregon Regional Primate Research Center (M.G.G., M.J.N.); Departments of Obstetrics and Gynecology, Legacy Emanuel Hospital and Health Center (M.G.G.); Oregon Health Sciences University (M.G.G., M.J.N.); Beaverton and Portland, Oregon*

### Abstract

Preterm birth remains the most common cause of perinatal mortality. Although the causes of preterm labor are multifactorial and vary according to gestational age, preterm labor and term labor share common cellular and molecular mechanisms, including stimulation of the fetal hypothalamic-pituitary-adrenal (HPA) axis and endocrine/immune system interactions. We have developed a non-human primate experimental model for intrauterine infection and preterm labor using chronically instrumented rhesus monkeys (*Macaca mulatta*) with timed gestations. We have documented the temporal and quantitative relationships among intrauterine infection, the synthesis and release of proinflammatory cytokines, prostaglandins, and fetal-placental steroid biosynthesis in this model. Infection-induced preterm parturition is characterized by significant elevations in amniotic fluid proinflammatory cytokines and by increases in fetal adrenal steroid biosynthesis, but not by corresponding increases in placental estrogen biosynthesis characteristic of spontaneous parturition. This suggests that activation of the fetal HPA axis by the stress of infection is accompanied by placental dysfunction and also that infection-induced preterm parturition is not dependent upon the increased estrogen biosynthesis observed in spontaneous parturition. These different endocrine and immune responses have important diagnostic and therapeutic implications in the management of preterm labor. Infect. Dis. Obstet. Gynecol. 5:142–153, 1997. © 1997 Wiley-Liss, Inc.

Key words
Intrauterine infection, steroidogenesis, cytokines, preterm labor

Preterm labor that results in premature birth is the most common cause of perinatal mortality in developed countries, accounting for up to 80% of perinatal deaths which are not attributable to congenital malformations.[1] Until recently, a tendency has existed among obstetricians and epidemiologists to lump together, for statistical purposes, all preterm births occurring between 22 and 36 weeks. The traditional empiric approach to preterm labor presupposed a single pathologic process, for which the treatment could be uniform. This practice has contributed to the poor understanding of the causation of preterm labor.

It is now clear that the causes of preterm labor are multifactorial and vary according to gestational age. They include systemic and intrauterine infection, uteroplacental ischemia, uterine overdistension, different endocrinopathies, and abnormal fetal or maternal immune responses.[2] Nevertheless, there is strong evidence that despite different etiologies, preterm labor and term labor share many common pathways in the activation of cellular and molecular effectors. This includes stimulation of the fetal hypothalamic-pituitary-adrenal (HPA) axis (e.g., by maturation, infection or fetal hypoxemia) in addition to endocrine, paracrine and immune system interactions.

We have developed the first nonhuman primate

Correspondence to: Dr. Michael G Gravett, Division of Reproductive Sciences, Oregon Regional Primate Research Center, 505 NW 185th Avenue, Beaverton, OR 97006 USA
Supported by NIH Grants AI42490, RR00163, HD-06159, and HD-18185

Received 1 October 1997

Accepted 21 October 1997

experimental model for chorioamnionitis and preterm labor using chronically catheterized pregnant rhesus monkeys (in which both placentation and the endocrinology of pregnancy are similar to humans). This model provides a useful means to study *in vivo* endocrine, paracrine and immune system interactions in the relatively inaccessible intrauterine space. This review will emphasize our findings with respect to the role of activation of the fetal HPA axis in response to stress associated with intrauterine infection and in comparison to that associated with spontaneous labor at term and idiopathic preterm labor. We will explore the interesting differences in activation of the fetal HPA axis and of the subsequent fetal-placental steroid biosynthesis in spontaneous parturition and in the setting of infection-induced preterm labor. These observations about different endocrine and immune responses may ultimately have important diagnostic and therapeutic implications in the management of preterm labor.

### REGULATION OF MYOMETRIAL QUIESCENCE

Parturition represents a sequential orchestration of specific biochemical and biophysical changes in the myometrium, decidua, fetal membranes and cervix. The mechanisms by which the transitions are made from uterine quiescence to the initiation of parturition and to the onset of labor are central to our understanding of primate and human parturition. Competing hypotheses include the release of uterotonins (e.g., estrogens, oxytocin, and prostaglandins) which overrides myometrial inhibition or a primary withdrawal or antagonism of progesterone's inhibitory effects. It is also not clear whether the transitional or initiating stimuli bring about amnion or decidual activation with subsequent cytokine and prostaglandin release.

We, and others, have demonstrated that parturition in rhesus monkeys is preceded by rising concentrations of dehydroepiandrosterone sulfate (DS) in fetal blood but not in maternal blood and by an increase in estrogens in fetal blood, maternal blood and amniotic fluid.[3,4] A rise in amniotic fluid estrone precedes or coincides with a rise in amniotic fluid prostaglandins which begin to increase several days prior to the onset of labor near term.[5] An increase in interleukin (IL)-6 and IL-8 (unpublished data), but not in IL-1β or tumor necrosis factor (TNF), is also seen several days prior to the onset of labor.[6] Suppression of prostaglandin biosynthesis by long-term administration of indomethacin during rhesus pregnancy dramatically prolongs gestation and prevents the timely onset of parturition.[7]

The stimulus for the rise in fetal and maternal estrogens before parturition has not been clearly defined but is likely to be fetal adrenocorticotropic hormone (ACTH), because hypoxemic stress or infusions of ACTH to the fetus increase production of androgens, such as DS and androstenedione.[8,9] A functioning fetal adrenal gland is clearly required because the prepartum estrogen trends are absent when the fetus is dead, anencephalic or dexamethasone suppressed; parturition is delayed in those cases.[7,9] Estradiol benzoate administered systemically to pregnant monkeys does not initiate labor before term, perhaps because exogenous estradiol does not cross the placenta to impact potential fetal target tissues.[7] Administration of androstenedione (an estrogen precursor) has led to conflicting results on initiation of premature delivery depending on dose and route of administration.[10,11] If estrogens facilitate parturition in primates, then stimulation of adrenal estrogen precursors by ACTH should induce premature labor. However, chronic infusion of ACTH into the primate fetus has led to conflicting results and more work needs to be done to confirm this hypothesis.[5,12]

Circadian rhythms in circulating maternal and fetal steroid hormones have also been described in pregnant nonhuman primates and women.[4,13] In the mother, cortisol, estrone and estradiol are higher in the morning than in the evening. Because the primate placenta is readily permeable to glucocorticoids, the fetal pituitary is subject to feedback regulation by maternal cortisol. As a result, there is a 180° phase difference between rhythms in fetal adrenal androgens and the maternal cortisol rhythm.[4] Estrogens enhance the oxidation of cortisol to cortisone in the placenta thereby decreasing the proportion of maternally-derived cortisol in the fetus.[14] This mechanism enhances ACTH secretion by the fetal pituitary and promotes the growth and maturation of the fetal adrenal cortex and *de novo* cortisol production as gestation advances.[15]

There is clear evidence that estrogens interact with the maternal circadian system to influence the timing of birth within the light-dark cycle. Studies in our laboratory[16] and by others[17] have clearly established a nocturnal increase in uterine activity which predominates in the last weeks of pregnancy and culminates in labor and delivery. In our view, rising estrogen concentrations during late rhesus pregnancy play a supportive role in establishing nocturnal uterine activity which is mediated by maternal, but not fetal, oxytocin.[16] Estrogen biosynthesis by the fetal-placental unit and subsequent maternal oxytocin release and heightened myometrial sensitivity to oxytocin play a major role in the transition from myometrial quiescence to uterine awakening.

In contrast to other mammalian species, progesterone concentrations do not decline prior to parturition in primates. Because a decline in progesterone is so ubiquitous in mammals, investigators have speculated that a local, intrauterine withdrawal of progesterone probably occurs in primates. Recent work has focused on mechanisms which may affect the local tissue withdrawal of progesterone at the level of fetal membranes or decidua while circulating progesterone levels remain elevated prepartum. Inhibition of the conversion of pregnenolone to progesterone by 3β-hydroxysteroid dehydrogenase inhibition mimics spontaneous labor and results in vaginal delivery suggesting that physiological progesterone withdrawal is a likely candidate to mediate the transition from uterine quiescence to labor.[18] However, we are left with a conundrum because pharmacologic progesterone withdrawal induces preterm labor and delivery (which can be blocked by progesterone substitution) but exogenous progesterone, even in substantial quantities, does not prevent parturition at term, possibly because it does not gain access to specific intrauterine tissue sites.[18] Alternatively, spontaneous labor may be triggered by a natural antiprogestin that appears at term and blocks progesterone action at a specific site.

### PATHOPHYSIOLOGY OF PRETERM LABOR

### Endocrine Mechanisms

Normally, spontaneous parturition in primates is preceded by increased fetal adrenal synthesis of C19 androgens and rising concentrations of DS and androstenedione in the fetal blood. In turn, these are converted by the placenta into estrogens whereby increasing concentrations of estradiol appear in maternal blood and rising estrone levels appear in maternal blood, fetal blood, and amniotic fluid. The rise in estrone precedes or coincides with the rise in amniotic fluid prostaglandins which also begin to increase several days prior to parturition.[4] Peripheral cortisol and progesterone concentrations remain relatively unchanged in the days preceding parturition but may increase at delivery. The primate placenta, like the human placenta contains steroid aromatase, but not 17α-hydroxylase activity. Thus, estrogen production during pregnancy is dependent upon an intact fetal-placental unit, with fetal adrenal C19 androgens as precursors to placentally-derived estrogens.

The role of estrogens in preterm delivery in humans is supported by the observation that maternal 17β-estradiol plasma concentrations are elevated 9 days prior to preterm labor in women who deliver 4–6 weeks prior to term.[19] Additionally, increases in salivary estriol concentrations have been detected 3 weeks prior to delivery among women delivering five weeks prematurely.[20] Clinical investigators have emphasized the potential importance of monitoring diurnal patterns of uterine activity throughout gestation as a screen for preterm birth.[19,21] By analogy with results from nonhuman primates, it is likely that an increase in estrogen biosynthesis and in nocturnal uterine activity often seen in human preterm parturition results from premature activation of the fetal HPA axis mediated by the release of ACTH and of placental corticotropin-releasing hormone (CRH) secondary to maternal stress, uteroplacental ischemia, or fetal hypoxemia (see Mesiano and Jaffe for a review).[22] The syncytiotrophoblast appears to be the major source of CRH as well as its binding protein during pregnancy. Corticotropin-releasing hormone has been noted to increase, and its binding protein to decrease, 6 weeks prior to term or preterm parturition.[23] Glucocorticoids, cytokines (especially IL-1) and prostaglandin $E_2$ ($PGE_2$) have stimulatory effects on placental CRH.

Recently, Karalis and colleagues presented evidence that cortisol reverses the inhibitory effects of progesterone on placental CRH by displacing progesterone from the glucocorticoid receptor.[24] Since CRH is secreted into both fetal and maternal circulations, it could (via stimulation of fetal pituitary ACTH and cortisol) complete a positive feedback loop that would be terminated by delivery. Another potentially important role for CRH in parturition arises from its vasodilatory and proinflammatory ef-

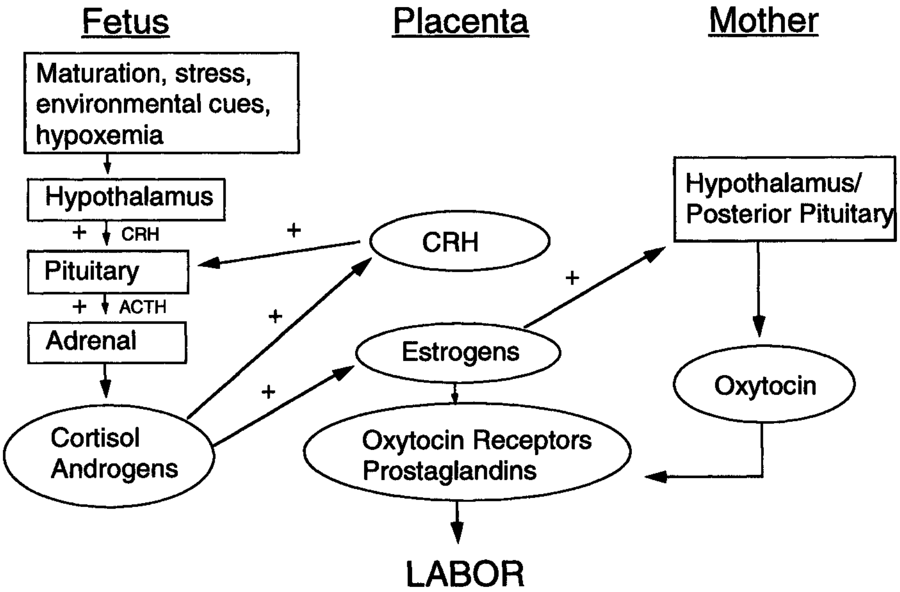

Fig. 1. A perspective on labor emphasizing endocrine mechanisms involving the fetal hypothalamic-pituitary-adrenal (HPA) axis and fetal-placental steroidogenesis. Increased placental corticotropin releasing hormone (CRH) secretion into the fetal circulation stimulates fetal ACTH production, which, in turn, stimulates cortisol and C19 androgen production (primarily dehydroepiandrosterone sulfate) by the fetal adrenal. Cortisol completes a positive feedback loop to further stimulate placental CRH secretion. Fetal adrenal androgens are converted to estrogens by the placenta. These estrogens then stimulate maternal oxytocin secretion, myometrial oxytocin receptors, myometrial gap junction formation, and prostaglandin production. These events are necessary to facilitate uterine contractions and labor and are likely also modulated by progesterone.

fects which include enhanced production of cytokines.[25] Thus, endocrine mechanisms which modulate preterm labor depend upon an intact fetal-placental unit for estrogen biosynthesis and activation of the fetal HPA axis with resultant fetal adrenal synthesis of C19 androgens and subsequent aromatization to C18 estrogens by the placenta (Figure 1). These mechanisms may contribute to a large proportion of preterm births occurring between 30 and 36 weeks of gestation, where preeclampsia, uteroplacental ischemia, intrauterine growth retardation and drug abuse predominate as contributory factors.

### Inflammatory/Infectious Mechanisms

We return now to the premise that microbial colonization and inflammation/infection in the maternal genital tract is another important cause of preterm birth, especially in those pregnancies that end in the second or early third trimester and result in very low birthweight neonates in whom mortality and morbidity are high. The prevalence of histologic chorioamnionitis is inversely related to gestational age and occurs in 60–90% of gestations ending between 20 and 24 weeks;[26,27] microbial infection of the chorioamnion occurs in 60% of patients with preterm delivery.[28]

Bacteria indigenous to the lower genital tract have been recovered from the amniotic fluid of 5–20% of women in preterm labor with intact fetal membranes.[2] The prevalence of amniotic fluid infection is also inversely related to gestational age, occurring in up to 67% of pregnancies ending at 23 to 24 weeks of gestation.[29] Furthermore, a high proportion of women in preterm labor with positive amniotic fluid cultures are refractory to standard tocolytic therapy and experience rapid preterm delivery (62.5% versus 13% of women with sterile amniotic fluid).[2] This suggests that the pathophysiology of preterm labor associated with intraamniotic infection is different from that of idiopathic preterm labor.

There is now considerable evidence to suggest that cytokines participate in the pathogenesis of infection-associated preterm labor. These inflammatory mediators include IL-1, IL-6, IL-8 and TNF and are produced by macrophages and decidual cells in response to a wide variety of bacteria

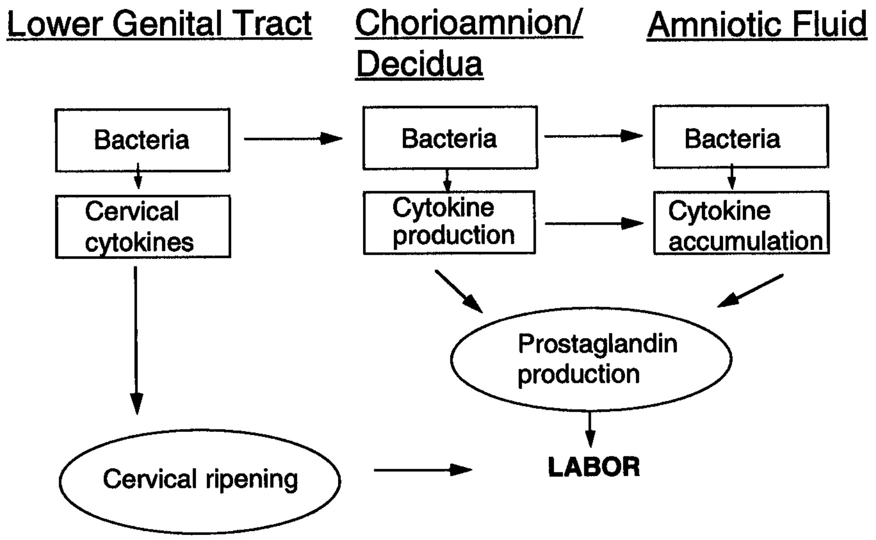

Fig. 2. A perspective on labor emphasizing infection and inflammation. Microorganisms from the lower genital tract transgress cervical barriers and gain entry into the choriodecidual space. Microorganisms within the cervix stimulate the local production of proinflammatory cytokines including IL-1, IL-6, IL-8 and TNF, which lead to neutrophilic infiltration, prostaglandin production and cervical softening and effacement. Microorganisms in the choriodecidual space leads to recruitment of maternal and fetal macrophages and neutrophils, release of proinflammator cytokines and prostaglandin or other eicosanoid production. Microorganisms may transgress the amnion and chorion, resulting in intraamniotic infection, activation of the fetal immune response and HPA axis, and amniotic fluid accumulation of proinflammatory cytokines and prostaglandins. Increase in prostaglandins, or other eicosanoids, may then stimulate myometrial contractions and labor.

or bacterial products. A role for selected cytokines in the onset of preterm parturition is based upon the following observations: (1) elevated concentrations of IL-1β, IL-6, and TNF and prostaglandins are found in the amniotic fluid of patients with intraamniotic infection and preterm labor;[30,31,32,33,34] (2) bacterial products stimulate the production of IL-1, IL-6, and TNF by human decidua;[35,36] (3) these cytokines, in turn, stimulate production of prostaglandins by *in-vitro* amnion and decidua;[33,37,38] and (4) systemic administration of recombinant IL-1 to pregnant mice induces preterm labor which can be prevented by pretreatment with IL-1 receptor antagonist protein.[39]

Thus, in the setting of infection the interaction between proinflammatory cytokines and prostaglandins occurring at the level of the chorioamnion and decidua is fundamentally important for preterm delivery (Figure 2).

### ENDOCRINE-IMMUNE INTERACTIONS

There is increasing evidence for important physiologic and molecular interactions between the immune system and the endocrine system, especially at the hypothalamic-pituitary and adrenal levels as well as locally in tissues.[40,41,42] From a teleological standpoint, such interactions are relevant considering the important functions of the adrenocortical system and the immune system in responding to stress. Some investigators have suggested that the response of the pituitary and adrenal glands to stress or infection may have evolved to suppress and modulate inflammatory responses.[41] It is noteworthy that IL-1, IL-2, IL-6 and TNF can augment the release of ACTH by stimulating hypothalamic CRH.[41,43] Interleukin-1 and IL-6 stimulate adrenocortical cells directly to release cortisol.[40,44] The adrenal gland is a major site of IL-6 production (which can be stimulated by ACTH, angiotension II, or cytokines such as IL-1) and which acts in an autocrine or paracrine manner to stimulate production of dehydroepiandrosterone and cortisol.[44] Therefore, IL-6 appears to play a role in integrating the adrenal responses to diverse endocrine and immune stimuli. It has been postulated that the acute regulation of the HPA axis by IL-6 is mediated via the hypothalamus, whereas at the level of the adrenal gland, IL-6 exerts its action in a more sustained fashion.[44] In turn glucocorticoids are well established immunosuppressants (in

TABLE 1. Steroid Hormone—Cytokine Interactions

| |
|---|
| Steroid Hormones |
| Glucocorticoids: inhibit IL-1, IL-2, IL-6, IL-8, TNF-α, IFN-γ (Th1 cytokines) enhance IL-4, IL-10 production (TH2 cytokines) |
| DHEA: inhibits TNF-α production |
| Progesterone: inhibits IL-8 and IFN-γ production |
| Estrogen: inhibits IL-1 production (>$10^{-7}$ Molar) enhances IL-1 production (<$10^{-9}$ Molar) |
| Cytokine |
| IL-1: stimulate trophoblast estrogen production |
| IL-1, TNF-α: stimulate trophoblast progesterone production |
| IL-1, IL-2, IL-6, TNF-α: stimulate hypothalamic CRH/corticotropin |
| IL-6: stimulate $C_{19}$androgens |

part by blocking the transcription factor NF-kB which is necessary for transcription of genes for inflammatory cytokines).[45] However, recent studies indicate a hierarchy of sensitivity, with TNF being most suppressible, IL-1 having intermediate sensitivity, and IL-6 being relatively resistant to glucocorticoid suppression.[46] Glucocorticoids also induce apoptosis of certain lymphocyte subpopulations, and shift the host T-cell response from the inflammatory Th1 type to the helper Th2 type (see Wick for review).[47] However, other steroid hormones elaborated within the fetal-placental unit may also modulate cytokine production and function (Table 1). High concentrations of estrogens or progesterone have been reported to inhibit spontaneous IL-1 production in monocytes.[48] Other roles for progesterone have been suggested, including immunosuppresant and antiinflammatory effects. Progesterone limits leukocytic infiltration in tissues and alters cytokine production (e.g., inhibits IL-8) which may have important effects on decidual activation and cervical ripening.[49] Dehydroepiandrosterone has also been demonstrated to reduce TNF production and protects mice from endotoxin toxicity.[50]

In view of the important interactions between the endocrine and immune systems alluded to above, we hypothesized that normal steroid biosynthesis by the fetal-placental unit may be altered or impaired in the setting of intrauterine infection. Therefore, we studied the temporal and quantitative relationships among intrauterine infection, cytokine and prostaglandin production, preterm labor, and fetal-placental steroid biosynthesis in a nonhuman primate model utilizing chronically catheterized rhesus monkeys (*Macaca mulatta*) with timed gestations, in which intrauterine infection was induced by the inoculation of known quantities of a specific microorganism.

### THE NONHUMAN PRIMATE MODEL IN INTRAUTERINE INFECTION

The chronically catheterized simian preparation provides a useful means to study the *in vivo* immune, endocrine, and even paracrine interactions in the relatively inaccessible intrauterine space. It offers several advantages over experimental models in lower mammalian species. Rhesus placentation is hemochorial and the endocrine events surrounding parturition are qualitatively similar to human pregnancy.[5,51] Samples of maternal and fetal blood and amniotic fluid can readily be obtained in a serial fashion from individual animals and uterine activity can be continuously monitored and correlated with indices of infection and inflammatory mediators.

In our model, on approximately day 110 of gestation (with term being 167 days), pregnant rhesus monkeys are conditioned to a jacket and tether system.[52] After conditioning, intrauterine surgery with implantation of maternal and fetal vascular catheters, open-ended intraamniotic pressure catheters, myometrial electromyographic electrodes, and fetal electrocardiographic electrodes is performed at or near 120 days gestation.[53] All animals receive terbutaline sulfate (1 mg intravenously over 3 to 5 hours, twice daily) for 1 to 5 days after surgery to control postoperative uterine irritability. Animals also receive cefazolin (250 mg intravenously, every 12 hours) which is discontinued at least 72 hours prior to infection or other experimentation.

### Experimental Intrauterine Infection

After postoperative stabilization for 10 days (approximately day 135 of gestation), we have reproducibly established intrauterine infection in pregnant rhesus monkeys by inoculation, through one of the intraamniotic pressure catheters, of $10^6$ colony forming units (cfu) of Group B *streptococci*, type III.[6] Other control animals were treated in a similar manner, but were not infected, and were followed to spontaneous parturition. Both before and after inoculation, amniotic fluid samples were collected serially from all animals for quantitative bacterial cultures, Gram stain of uncentrifuged

TABLE 2. Summary of amniotic fluid bacterial counts, cytokines and prostaglandin concentrations, and uterine activity in rhesus monkeys (n = 4) with experimental intraaminiotic infection*

| | Event | | | |
|---|---|---|---|---|
| | Preinoculation | Inoculation | Onset Contractions | Delivery |
| GBS (cfu/ml)[a] | n.g. | $5 \times 10^6$ | $8 \times 10^9$ | $1 \times 10^{10}$ |
| HCA (mmHg sec/h)[b] | 100 | 150 | 4,600 | 12,500 |
| TNF (pg/ml) | 52 | 29 | >20,000 | >20,000 |
| IL-1β (pg/ml) | <20 | <20 | 1,504 | 2,467 |
| IL-6 (ng/ml) | 12.9 | 9.9 | 49.6 | 46 |
| $PGE_2$ (pg/ml) | 494 | 510 | 16,046 | 15,274 |
| $PGF_{2\alpha}$ (pg/ml) | 215 | 280 | 5,547 | 5,490 |

*Expressed as median values.
[a]Geometric mean titer.
[b]HCA: area under the amniotic fluid pressure curve, expressed as mean hourly contractions area.

fluid, white blood cell analysis by hemocytometer, cytokine (IL-1β, IL-6 and TNF) concentrations were determined by enzyme-linked immunoassay (EIA) or bioassay and prostaglandin ($PGE_2$ and $PGF_{2a}$) concentrations were determined by EIA. Fetal and maternal blood were also serially sampled for a complete blood cell analysis and for sex steroid hormone concentrations. The fetal electrocardiogram and uterine activity (by electromyelography and intraamniotic pressure) were continuously recorded from the time of surgery until delivery. The uterine contractility was recorded as the area under the contraction curve per hour and expressed as the hourly contraction area (HCA) in mmHg seconds/hour. Consistency, effacement, and dilatation of the maternal cervix, and maternal rectal temperature were also monitored.

Following intraamniotic inoculation with $10^6$ Group B *streptococci* there was rapid exponential growth within amniotic fluid. Associated with this infection, there were sequential increases in amniotic fluid cytokines, prostaglandins, and uterine activity (from pre-inoculation HCA levels of 0 to 100 mmHg sec/h to peak levels of 10,000 to 20,000 mmHg sec/h at a mean of 28 hours (range 14–40 hours) after inoculation), as summarized in Table 2. The marked increase in uterine contractility led to progressive cervical effacement and dilatation in infected animals. In all infected animals, increases in uterine activity was accompanied by cervical dilatation and/or effacement. All infected animals were delivered within 72 hours of infection. In contrast, animals followed as a control group progressed to spontaneous parturition 30 to 45 days following surgery and all delivered near term (167 days).

Amniotic fluid concentrations of TNF, IL-1β and IL-6 also rose dramatically following experimental intraamniotic infection and prior to increases in uterine contractility as shown in Table 2 and as we have previously reported.[6] Amniotic fluid TNF concentrations rose an average of 9 hours (range 6–14 hours) after inoculation and 20 hours (range 8–34 hours) before increases in uterine contractility. Increases in IL-1β and in IL-6 occurred 15 to 18 hours after inoculation, and preceded increases in uterine contractility by 10 to 13 hours. Increases in amniotic fluid TNF preceded increases in amniotic fluid IL-1β or IL-6 concentrations by 4 to 8 hours in all infected animals and represented the earliest marker of infection in our study. The elevations in amniotic fluid cytokines observed after experimental infection are consistent with those reported in pregnant women with infection-associated labor.[30,32,33,34]

In contrast to preterm labor induced by intraamniotic infection in our model, spontaneous parturition near term in control animals was not associated with increases in amniotic fluid concentrations of either IL-1β or TNF above low basal levels, and only modest increases in IL-6 were observed.[6]

Following intraamniotic infection, increases in amniotic fluid $PGE_2$ and $PGF_{2a}$ occurred in parallel with elevations in amniotic fluid cytokines and preceded increases in uterine contractility. The most striking increase was in $PGE_2$, which rises to concentrations in excess of 15,000 pg/ml at delivery (Table 2). Similar, but smaller increases in $PGF_{2a}$ also occur. These results are consistent with human *in-vitro* studies which have demonstrated that fetal membrane, decidual, and placental production

TABLE 3. Median steroid hormone concentrations among rhesus monkeys (n = 4) in spontaneous parturition near term

| | Compartment | | | | | |
|---|---|---|---|---|---|---|
| | Fetal Artery | | Maternal Artery | | Amniotic Fluid | |
| | Basal | Delivery | Basal | Delivery | Basal | Delivery |
| Estrogens | | | | | | |
| Estrone (pg/ml) | 223 | 805* | 314 | 520* | 115 | 706* |
| Estradiol (pg/ml) | 33 | 120* | 378 | 579* | 8 | 63* |
| Androgens | | | | | | |
| Androstenedione (ng/ml) | 0.98 | 4.39* | 0.71 | 1.18 | 0.63 | 0.73 |
| Dehydroepiandrosterone (ng/ml) | 11.42 | 17.77* | 22.8 | 28.8 | 3.22 | 11.0* |
| Dehydroepiandrosterone sulfate (ng/ml) | 338 | 3,083* | 357 | 367 | 51 | 159 |
| Glucocorticoids | | | | | | |
| Cortisol (ng/ml) | 89 | 138 | 204 | 237 | 107 | 150 |
| Progesterone (ng/ml) | 2.8 | 15.6 | 3.2 | 5.4* | 0.10 | 0.21* |

Basal concentrations were determined from samples obtained 14 to 24 days prior to parturition. Delivery concentrations were identified as the last sample taken (no more than 1 day prior to delivery). *$p < 0.05$, Mann-Whitney U test. Data presented as median. Adapted from Gravett,[56].

rates of $PGE_2$ and $PGF_{2a}$ are higher in the presence of histologic chorioamnionitis.[54,55]

### Fetoplacental Steroidogenesis in Experimental Intrauterine Infection

To investigate the complex interplay of both positive and negative feedback effects among the various cytokines, prostaglandins, and steroid hormones (as reviewed above), we also measured and compared, by specific RIA, adrenal androgens (androstenedione, dehydroepiandrosterone, and dehydroepiandrosterone sulfate), estrogens (estrone and estradiol), progesterone, and cortisol concentrations in rhesus monkeys with experimental intrauterine infection and in monkeys delivered normally at term.[56]

Spontaneous parturition was characterized by an initial increase in fetal plasma concentrations of progesterone (P) and the adrenal androgens, androstenedione (A), dehydroepiandrosterone (D), and dehydroepiandrosterone sulfate (DS) which occurred 8 to 10 days prior to the onset of labor. These changes are summarized in Table 3. Increases in placental estrogen biosynthesis followed increases in fetal adrenal androgen production, beginning 6 to 8 days before spontaneous parturition. Although there was a tendency for the concentration of cortisol to increase in fetal plasma and in amniotic fluid, these changes were not statistically significant.

In contrast to spontaneous parturition, preterm labor induced by intrauterine infection was characterized by abrupt increases in fetal adrenal androgen and cortisol production, but without corresponding increases in placental estrogen biosynthesis, as summarized in Table 4. In all instances, increases in fetal adrenal steroid biosynthesis occurred concurrently with or after increases in uterine contractility. Progesterone (P) increased significantly in the fetal artery, the maternal artery, and in amniotic fluid, which reflects in part increased adrenal production of pregnenolone and conversion to progesterone by the placenta. Significant increases in androstenedione (A) were also observed in all three compartments. Dehydroepiandrosterone (D) increased in the fetal artery and in amniotic fluid; dehydroepiandrosterone sulfate (DS) increased in the fetal artery.

Despite an increase in fetal adrenal androgen production, no parallel increases in placental estrogen biosynthesis were observed in the setting of infection-induced preterm labor (Table 4). Only a minimal increase in amniotic fluid estrone (E1), from 34 pg/ml prior to infection to 114 pg/ml at delivery, was observed. There were no changes in the concentration of estradiol (E2) in either the fetal or maternal compartment. Intense stimulation of the fetal HPA axis in the setting of infection-induced labor was confirmed by significant increases in fetal concentrations of cortisol which increased from 91 ng/ml prior to infection to 221 ng/ml by delivery (Table 4).

### COMMENT

Since the endocrine changes observed during infection-induced parturition occur with or soon after

TABLE 4. Median steroid hormone concentrations in rhesus monkeys (n = 6) with infection-induced preterm parturition

| | Compartment | | | | | |
|---|---|---|---|---|---|---|
| | Fetal Artery | | Maternal Artery | | Amniotic Fluid | |
| | Basal | Delivery | Basal | Delivery | Basal | Delivery |
| Estrogens | | | | | | |
| Estrone (pg/ml) | 156 | 170 | 147 | 172 | 34 | 114* |
| Estradiol (pg/ml) | 26 | 39 | 277 | 279 | 7.75 | 24.5 |
| Androgens | | | | | | |
| Androstenedione (ng/ml) | 0.96 | 2.44* | 0.64 | 1.00* | 0.08 | 0.29* |
| Dehydroepiandrosterone (ng/ml) | 8.81 | 20.24* | 23.02 | 39.48 | 2.35 | 5.61* |
| Dehydroepiandrosterone sulfate (ng/ml) | 284 | 850* | 188 | 189 | 18.5 | 22.9 |
| Glucocorticoids | | | | | | |
| Cortisol (ng/ml) | 91 | 221* | 257 | 349 | 112 | 162 |
| Progesterone (ng/ml) | 2.58 | 8.75* | 2.56 | 5.53* | 0.04 | 0.20* |

Basal concentrations were determined from samples obtained for several days prior to infection. Delivery concentrations were identified as the last sample taken prior to delivery. *$p < 0.05$, Mann-Whitney U test. Data presented as median. Adapted from Gravett,[56].

the onset of contractions, it is unlikely that they play a major role in the initiation of preterm labor in the setting of infection. Nevertheless, selected steroid hormones may modulate immune function in the amniotic cavity and fetal membranes (by analogy with the adrenal gland or other hormone responsive tissues).[42,44] While we did not measure ACTH or catechols in our study, the fetal endocrine profiles which we have observed during intrauterine infection are consistent with acute fetal stress and resultant activation of the fetal pituitary-adrenal axis.[8,57] It is not clear to what extent stimulation of the fetal adrenal gland is due to ACTH secretion secondary to fetal hypoxemia or to cytokine-mediated effects. Although we did not observe signs of severe fetal hypoxemia (late decelerations of the fetal heart rate were absent during labor), moderate fetal hypoxemia is likely, since fetuses were bacteremic or had congenital pneumonia at birth.

Previous studies in our laboratory have demonstrated that the extent of placental clearance of androgens through estrogen formation is proportional to uteroplacental blood flow[58] and that maternal circulating estradiol concentrations in response to reduced blood flow vary with the extent of fetal hypoxemia.[59] We have also demonstrated that elevated maternal and fetal estrogen concentrations after acute fetal hypoxemia result from placental conversion of an augmented supply of fetal androgens.[8] Thus, we were surprised in our current work with intrauterine infection that an augmented estrogen response was absent in both the maternal and fetal circulations despite an increase in fetal androstenedione, D, and DS. This suggested to us selective placental dysfunction, since progesterone production actually increased.

The reasons for the apparent placental dysfunction remain speculative but may include cytotoxic effects of infection on the placenta or fetal membranes, diminished placental blood flow, relative hypoxia, or deficient conversion of androgens to estrogens secondary to the effects of proinflammatory cytokines or their byproducts on the aromatase cytochrome P-450 enzyme system. In the presence of infection, abnormal placental metabolism and cytochrome P-450 dysfunction may be mediated by toxic lipid peroxides which are linked with cytokine activity, or by over production of nitric oxide and free radicals in placental villi.[60] Masuda et al. have reported that nitric oxide inhibits estradiol synthesis by inhibiting cytochrome P-450 aromatase.[61] It is possible that the aromatase complex which contains a specific cytochrome P-450 heme protein is more susceptible to these toxic effects than are non-P-450 enzymes such as 3-β-hydroxysteroid dehydrogenase and isomerase responsible for progesterone synthesis.

In summary, although there is evidence for activation of the fetal HPA axis in the setting of infection-induced preterm labor, many important questions remain. Are the adrenal effects the result of stress-induced CRH/ACTH stimulation or are they the result of direct action of proinflammatory cytokines on the fetal adrenal (or a combination of both)? Since CRH production by the placenta is modulated differentially by glucocorticoids and by progesterone, and since both hormones are elevated with intrauterine infection, the net effects on placental CRH production in the presence of infection remain to be determined. A further conundrum arises when one attempts to predict the effects of increased fetal cortisol on myometrial activity. While an augmented supply of glucocorticoids may down-regulate the inflammatory response, it may also serve to stimulate placental CRH production.[62] Among other actions, CRH would tend to increase uterine contractility by stimulating the release of prostaglandins and by sensitizing the myometrium to oxytocin.[63]

In the presence of infection, activation of the fetal HPA axis, resulting in an augmented supply of fetal androgens and aromatization to estrogens would increase the local estrogen/progesterone ratio and prepare the uterus for parturition by increasing oxytocin receptor density (by stimulating oxytocin gene expression in the chorion and decidua).[64] On the other hand, if estrogens are not increased, as we have demonstrated in the setting of intrauterine infection, labor proceeds by alternative mechanisms involving the proinflammatory cytokine/prostaglandin cascade.

If confirmed in human pregnancy, these fundamental differences in the pathophysiology of preterm labor may have profound implications for the diagnosis and management of preterm labor. For instance, salivary estriol has been demonstrated to be elevated among patients with idiopathic preterm labor.[20] Based upon our observations, we would not expect salivary estriol to be elevated among patients with infection-induced preterm labor. It is possible, therefore, that salivary estriol, alone or in combination with other tests, could, be utilized as a non-invasive test to distinguish between those patients with and without intrauterine infection.

The recognition of the multifactorial etiologies and different pathophysiologic mechanisms of preterm labor also has therapeutic implications. Since estrogens serve to up-regulate maternal oxytocin secretion and myometrial oxytocin receptors, oxytocin antagonists may be more useful in treating preterm labor not associated with infection. It is likely that comprehensive therapy for infection-induced preterm labor will include administration of a general tocolytic agent, the use of cyclooxygenase inhibitors to reduce eicosanoid effects, immunomodulators to counteract cytokine-induced inflammatory effects, and broad-spectrum antibiotics to eradicate specific microorganisms. Undoubtedly, in the near future, other endocrine, paracrine, and immune interactions will be elucidated which will effect the management of preterm labor. Our nonhuman primate model is well suited to study these interactions and to facilitate the development of logical intervention strategies.

### REFERENCES

1. Rush RW, Keirse MJ, Howat P, Baum JD, Anderson ABM, Trunbull AC: Contribution of preterm delivery to perinatal mortality. Br J Med 2:965–968, 1976.
2. Romero R, Avila C, Brekus CA, Morotti R: The role of systemic and intrauterine infection in preterm parturition. Ann NY Acad Sci 622:355–375, 1991.
3. Siiteri PK, Seron-Ferre M: Some new thoughts on the fetoplacental unit and parturition in primates. In: Novy MJ, Resko JA (eds.): Fetal Endocrinology, New York: Academic Press, pp. 1–34, 1981.
4. Walsh SW, Stancyzyk FZ, Novy MJ: Daily hormonal changes in the maternal, fetal, and amniotic fluid compartments before parturition in a primate species. J Clin Endocrinol Metab 58:629–639, 1984.
5. Novy MJ, Haluska GJ: Endocrine and paracrine control of parturition in rhesus monkeys. In: McNellis D, Challis JRG, Macdonald PC, Nathanielsz PW, Roberts JM, (eds): The Onset of Labor: Cellular and Integrative Mechanisms. New York: Perinatology Press, p. 321–334, 1988.
6. Gravett MG, Witkin SS, Haluska GJ, Edwards JL, Cook MJ, Novy MJ: An experimental model for intraamniotic infection and preterm labor in rhesus monkeys. Am J Obstet Gynecol 171:1660–1667, 1994.
7. Novy MJ, Walsh SW: Dexamethasone and estradiol treatment in pregnant rhesus macaques: Effects on gestational length, maternal plasma hormones, and fetal growth. Am J Obstet Gynecol 145:920–931, 1983.
8. Shepherd RW, Stanczyk FZ, Bethea CL, Novy MJ: Fetal and maternal endocrine responses to reduced uteroplacental blood flow. J Clin Endocrinol Metab 75:301–307, 1992.
9. Novy MJ, Haluska GJ: New perspectives on estrogen, progesterone and oxytocin action in primate parturition. In: Chwalisz K, Garfield RE (eds.): Basic Mechanisms Controlling Term and Preterm Birth. Heidelberg: Springer-Verlag, pp. 163–195, 1994.
10. Mecenas CA, Giusanni DA, Owiny JR, et al.: Production of premature delivery in pregnant rhesus monkeys by androstenedione infusion. Nat Med 2:443–448, 1996.
11. Haluska GJ, Cook MJ, Novy MJ: Subcutaneous administration of androstenedione increases maternal estradiol concentrations but does not lead to preterm labor and delivery in rhesus monkeys. 44th Annual Meeting of the Society for Gynecologic Investigation. San Diego, CA, March 19–22, 1997 (p. 86A, abstract 030).

12. Novy MJ: Endocrine and pharmacological factors which infuence the onset of labor in rhesus monkeys. In: O'Connor M, Knight J (eds.): The Fetus and Birth. New York: Elsevier, pp. 259–295, 1977.
13. Challis JRG, Sprague C, Patrick JE: Relation between diurnal changes in peripheral plasma progesterone, cortisol, and estriol in normal women at 30–31, 34–35, and 28–29 weeks of gestation. Gynecol Obstet Invest 16:33–44, 1983.
14. Pepe GJ, Waddell BJ, Stahl SJ, Albrecht ED: The regulation of transplacental cortisol-cortisone metabolism by estrogen in pregnant baboons. Endocrinology 122:78–83, 1988.
15. Pepe GJ, Waddell BJ, Albrecht ED: Activation of the baboon fetal hypothalamic-pituitary-adrenocortical axis at midgestation by estrogen-induced changes in placental corticosteroid metabolism. Endocrinology 127:3117–3123, 1990.
16. Hirst JJ, Haluska GJ, Cook MJ, Novy JM: Plasma oxytocin and nocturnal uterine activity: Maternal but not fetal concentrations increase progressively during late pregnancy and delivery in rhesus monkeys. Am J Obstet Gynecol 169:414–422, 1993.
17. Honnebier MBOM, Figueroa JP, Rivier J, Vale W, Nathanielsz PW: Studies of the role of oxytocin in late pregnancy in the pregnant rhesus monkey: plasma concentrations of oxytocin in the maternal circulation throughout the 24-h day and the effect of the synthetic oxytocin antagonist (1-B-Mpa (B-(CH2)5)1, (Me(Tyr2, Orn8) oxytocin. J Dev Physiol 12:225–232, 1989.
18. Haluska GJ, Cook MJ, Novy MJ: Inhibition and augmentation of progesterone production during pregnancy: Effects on parturition in rhesus monkeys. Am J Obstet Gynecol 176:682–691, 1997.
19. Germain AM, Kato S, Villarroel LA, Valenzuela GJ, Seron-Ferre M: Human term and preterm delivery is preceded by a rise in maternal plasma 17β-estradiol. Prenat Neonat Med 1:57–63, 1996.
20. McGregor JA, Jackson GM, Lachelin G, et al.: Salivary estriol as risk assessment for preterm labor: A prospective trial. Am J Obstet Gynecol 173:1337–1342, 1995.
21. Cooperstock M, England JE, Wolfe RA: Circadian incidence of labor onset hour in preterm labor and chorioamnionitis. Obstet Gynecol 70:852–855, 1987.
22. Mesiano S, Jaffe RB: Developmental and functional biology of the primate fetal adrenal cortex. Endocrine Rev 18:378–403, 1997.
23. McLean M, Bisits A, Davies J, Woods R, Lowry P, Smith R: A placental clock controlling the length of human pregnancy. Nat Med 1:460–463, 1995.
24. Karalis K, Goodwin G, Majzoub JA: Cortisol blockade of progesterone: A possible molecular mechanism involved in the initation of human labor. Nat Med 2:556–560, 1996.
25. Varnakoupolis NC, Chrousos GP. Hormonal regulation of human corticotropin-releasing hormone gene expression: Implications for the stress response and immune/inflammatory reaction. Endocrine Rev 15:409–420, 1994.
26. Russell P: Inflammatory lesions of the human placenta: I. Clinical significances of acute chorioamnionitis. Am J Diagn Gynecol Obstet 1:127–137, 1979.
27. Mueller-Heubach E, Rubinstein DN, Schwarz SS: Histologic chorioamnionitis and preterm delivery in different patient populations. Obstet Gynecol 75:622–626, 1990.
28. Hillier SL, Martius J, Krohn MA, Kiviat NB, Holmes KK, Eschenbach DA: A case-control study of chorioamnionitis in prematurity. N Engl J Med 319:972–978, 1988.
29. Watts DH, Krohn MA, Hillier SL, Eschenbach DA. The association of occult amniotic fluid infection with gestational age and neonatal outcome among women in preterm labor. Obstet Gynecol 79:351–357, 1992.
30. Hillier SL, Witkin SS, Krohn MA, Watts DH, Kiviat NB, Eschenbach DA: The relationship of amniotic fluid cytokines and preterm delivery, amniotic fluid infection, histologic chorioamnionitis, and chorioamnion infection. Obstet Gynecol 81:941–948, 1993.
31. Romero R, Emamian M, Wan M, Quintero R, Hobbins JC, Mitchell MD: Prostaglandin concentrations in amniotic fluid of women with intra-amniotic infection and preterm labor. Am J Obstet Gynecol 157:1461–1467, 1987.
32. Romero R, Brody DT, Oyarzun E, et al.: Infection and labor. III. Interleukin-1: A signal for the onset of parturition. Am J Obstet Gynecol 160:1117–1123, 1989.
33. Romero R, Manogue KR, Mitchell MD, et al.: Infection and labor. IV. Cachetin-tumor necrosis factor in the amniotic fluid of women with intraamniotic infection and preterm labor. Am J Obstet Gynecol 161:336–341, 1989.
34. Romero R, Avila C, Santhanam U, Sehgal PG: Amniotic fluid interleukin-6 in preterm labor. Association with labor. J Clin Invest 85:1392–1400, 1990.
35. Casey ML, Cox SM, Beutler B, Milewich L, MacDonald PC: Cachectin/tumor necrosis factor-alpha formation in human decidua. Potential role for cytokines in infection-induced preterm labor. J Clin Invest 83:432–436, 1989.
36. Romero R, Wu YK, Brody DT, Oyarzun E, Duff GW, Durum SK: Human dedcidua: A source of interleukin-1. Obstet Gynecol 73:31–34, 1989.
37. Mitchell MD, Dudley DJ, Edwin SS, Schiller Sl: Interleukin-6 stimulates prostaglandin production by human amnion and decidual cells. Eur J Pharmacol 192:189–191, 1991.
38. Romero R, Durum S, Dinarellow CA, Oyarzun E, Hobbins JC, Mitchell MD: Interleukin-1 stimulated prostaglandin biosynthesis by human amnion. Prostaglandins 37:13–22, 1989.
39. Romero R, Mazur M, Tartakovsky B: Systemic administration of interleukin-1 induces preterm parturition in mice. Am J Obstet Gynecol 165:969–971, 1991.
40. Aoki N, Ohno Y, Imamura M: Physiological interactions between the immune and endocrine systems: Are cytokines hormones? Med Sci Res 18:195–201, 1990.
41. Riechlin S: Neuroendrocrine-immune interactions. N Engl J Med 329:1246–53, 1993.
42. Besedovsky HO, Del Rey A: Immune-neuro-endocrine interactions: Facts and hypotheses. Endocrine Rev 17: 64–102, 1996.
43. Sweep F, Rijnkesl C, Hermus A: Activation of the hypothalamus-pituitary-adrenal axis by cytokines. Acta Endocrinologica 125:84–91,1991.
44. Path G, Bornstein SR, Ehrhart-Bornstein M, Scherbaum WA: Interleukin-6 and interleukin-6 receptor in the human adrenal gland: expression and effects on steroidogenesis. J Clin Endocrinol Metab 82:2343–2349, 1997.
45. Auphan N, DiDonato JA, Rosette C, Helmberg A, Karin M: Immunosupression by glucocorticoids: Inhibition of NF-$_k$ B synthesis. Science 270:286–280, 1995.
46. DeRijk R, Michelson D, Kapp B, et al.: Exercise and circadian rhythm-induced variations in plasma cortisol differentially regulate interleukin-1β (IL-1β), IL-6, and tumor necrosis factor-α (TNFα) production in humans: High sensitivity of TNFα and resistance of IL-6. J Clin Endocrinol Metab 82:2182–2191, 1997.
47. Wick G, Hu Y, Schwarz S, Kroemer G: Immunoendocrine communiation via the hypothalamo-pituitary-adrenal axis in autoimmune diseases. Endrocrine Rev 14:539–563, 1993.
48. Polan ML, Damele A, Kuo A: Gonadal steroids modulate human monocyte interleukin-1 (IL-1) activity. Fertil Steril 49:964–968, 1988.
49. Kelly RW: Pregnancy maintainance and parturition: the role of prostaglandin in manipulating the immune and inflammatory response. Endocrine Rev 15:684–706, 1994.
50. Dannenberg HD, Alpert G, Lustig S, Ben-Nathan D: Dehydroepiandrosterone protects mice from endotoxin toxicity and reduces tumor necrosis factor production. Antimicrob Agents Chemother 36:2275–2279, 1992.
51. Challis JRG, Olson DM: Parturition. In: Knobil E. Neill J, et al. (eds.): The Physiology of Reproduction. New York: Raven Press, Ltd, pp. 2177–2216, 1988.
52. Ducsay CA, Cook MJ, Novy MJ: Simplified vest and tether system for maintenance of chronically catheterized rhesus monkeys. Lab Anim Sci 38:343–344, 1988.
53. Haluska GJ, Stanczyk FZ, Cook MJ, Novy MJ: Temporal changes in uterine activity and prostaglandin response to RU486 in rhesus macaques in late gestation. Am J Obstet Gynecol 157:1487–1495, 1987.
54. Bernal AL, Hansell DJ, Khong TJ, Keeling JW: Prostaglandin E production by the fetal membranes in unexplained preterm labour and preterm labour associated with chorioamnionitis. Br J Obstet Gynecol 96:1133–1139, 1989.
55. Van der Elst CW, Bernal AL, Sinclair-Smith CC: The role of chorioamnionitis and prostaglandins in preterm labor. Obstet Gynecol 77:672–676, 1991.
56. Gravett MG, Haluska GJ, Cook MJ, Novy MJ: Fetal and maternal endocrine responses to experimental intrauterine infection in rhesus monkeys. Am J Obstet Gynecol 174:1725–1733, 1996.
57. Walsh SW, Nouman RL, Novy MJ: In utero regulation of rhesus monkey fetal adrenals.: Effects of dexamethasone, adrenocorticotropin, thyrotropin-releasing hormone, prolactin, human chorionic gonadotropin, and α-melonocyte-stimulating hormone on fetal and maternal plasma steroids. Endocrinology 104:1805-1813, 1979.
58. Fritz MA, Stanczyk FZ, Novy MJ: Relationship of uteroplacental blood flow to the placental clearance of maternal dehydroepiandrosterone through estradiol formation in the pregnant baboon. J Clin Endocrinol Metab 61:1023–1030, 1985.
59. Fritz MA, Stanczyk FZ, Novy MJ: Maternal estradiol response to alterations in uteroplacental blood flow. Am J Obstet Gynecol 155:1317–1325, 1986.
60. Myatt L, Brockman DE, Eis ALW, Pollack JS: Immunohistochemical localization of nitric oxide synthase in the human placenta. Placenta14:487–495, 1993.
61. Masuda M, Kubota T, Kamada S, Aso T: Nitric oxide inhibits steroidogenesis in cultured porcine granulosa cells. Molecular Human Reproduction 3:4 285–292, 1997.
62. Riley SC, Challis JRG: Corticotrophin-releasing hormone production by the placenta and fetal membranes. Placenta 12:105–119, 1991.
63. Quartero HWP, Fry CH: Placental corticotropin–releasing factor may modulate human parturition. Placenta 10:439–443, 1989.
64. Chibbar R, Wong S, Miller FD, Mitchell BF: Estrogen stimulates oxytocin gene expression in human choriodecidua. J Clin Endocrinol Metab 80:567–572, 1995
